# Aloe-emodin inhibits African swine fever virus replication by promoting apoptosis via regulating NF-κB signaling pathway

**DOI:** 10.1186/s12985-023-02126-8

**Published:** 2023-07-19

**Authors:** Yizhuo Luo, Yunlong Yang, Wenru Wang, Qi Gao, Ting Gong, Yongzhi Feng, Dongdong Wu, Xiaoyu Zheng, Guihong Zhang, Heng Wang

**Affiliations:** 1 Key Laboratory of Zoonosis Prevention and Control of Guangdong Province, College of Veterinary Medicine, South China Agricultural University, Guangzhou, 510462 China; 2 African Swine Fever Regional Laboratory of China (Guangzhou), Guangzhou, 510642 China; 3 Key Laboratory of Animal Vaccine Development, Ministry of Agriculture and Rural Affairs, Guangzhou, PR China; 4 Maoming Branch, Guangdong Laboratory for Lingnan Modern Agriculture, Maoming, 525000 China; 5 Research Center for African Swine Fever Prevention and Control, South China Agricultural University, Guangzhou, 510642 China

**Keywords:** African swine fever virus, Aloe-emodin, NF-κB signaling pathway, Apoptosis

## Abstract

African swine fever (ASF) is an acute infectious haemorrhagic fever of pigs caused by African swine fever virus (ASFV). Aloe-emodin (Ae) is an active ingredient of Chinese herbs with antiviral, anticancer, and anti-inflammatory effects. We investigated the antiviral activity and mechanism of action of Ae against ASFV using Real-time quantitative PCR (qPCR), western blotting, and indirect immunofluorescence assays. Ae significantly inhibited ASFV replication. Furthermore, transcriptomic analysis revealed that ASFV infection activated the NF-κB signaling pathway in the early stage and the apoptosis pathway in the late stage. Ae significantly downregulated the expression levels of MyD88, phosphor-NF-κB p65, and pIκB proteins as well as the mRNA levels of IL-1β and IL-8 in porcine alveolar macrophages (PAMs) infected with ASFV, thereby inhibiting the activation of the NF-κB signaling pathway induced by ASFV. Flow cytometry and western blot analysis revealed that Ae significantly increased the percentage of ASFV-induced apoptotic cells. Additionally, Ae promoted apoptosis by upregulating the expression levels of cleaved-caspase3 and Bax proteins and downregulating the expression levels of Bcl-2 proteins. This suggests that Ae promotes apoptosis by inhibiting the NF-κB pathway, resulting in inhibition of ASFV replication. These findings have further improved therapeutic reserves for the prevention and treatment of ASF.

## Introduction

African swine fever (ASF) is an acute infectious haemorrhagic fever of pigs caused by African swine fever virus (ASFV), which is listed by the World Organization for Animal Health (OIE) as a class of animal diseases. Domestic pigs and wild boars of all breeds and ages are susceptible to infection, with a fatality rate of up to 100% [1]. The clinical features of ASF include high fever, systemic hemorrhage, and splenomegaly [2, 3]. ASFV was firstly isolated and reported in Kenya in 1921, and subsequently spread to many countries and regions worldwide [4]. ASFV belongs to the *Asfarviridae* family and is the only known double-stranded DNA arbovirus [5]. The genome of ASFV is approximately 170–193 kb and contains more than 150 open reading frames (ORFs), encoding 150–200 proteins [6]. ASFV uses a variety of mechanisms to suppress and evade host immune responses, thereby promoting its replication [7]. The first ASF outbreak was reported in China in August 2018, and the subsequent spread of the epidemic caused severe economic losses to China’s pig industry [8]. ASFV-related vaccines have been developed in many countries in recent years; however, their protection and safety profile remains controversial [9]. Therefore, the development of effective anti-ASFV drugs is important for the prevention and treatment of ASF.

**Fig. 1:**
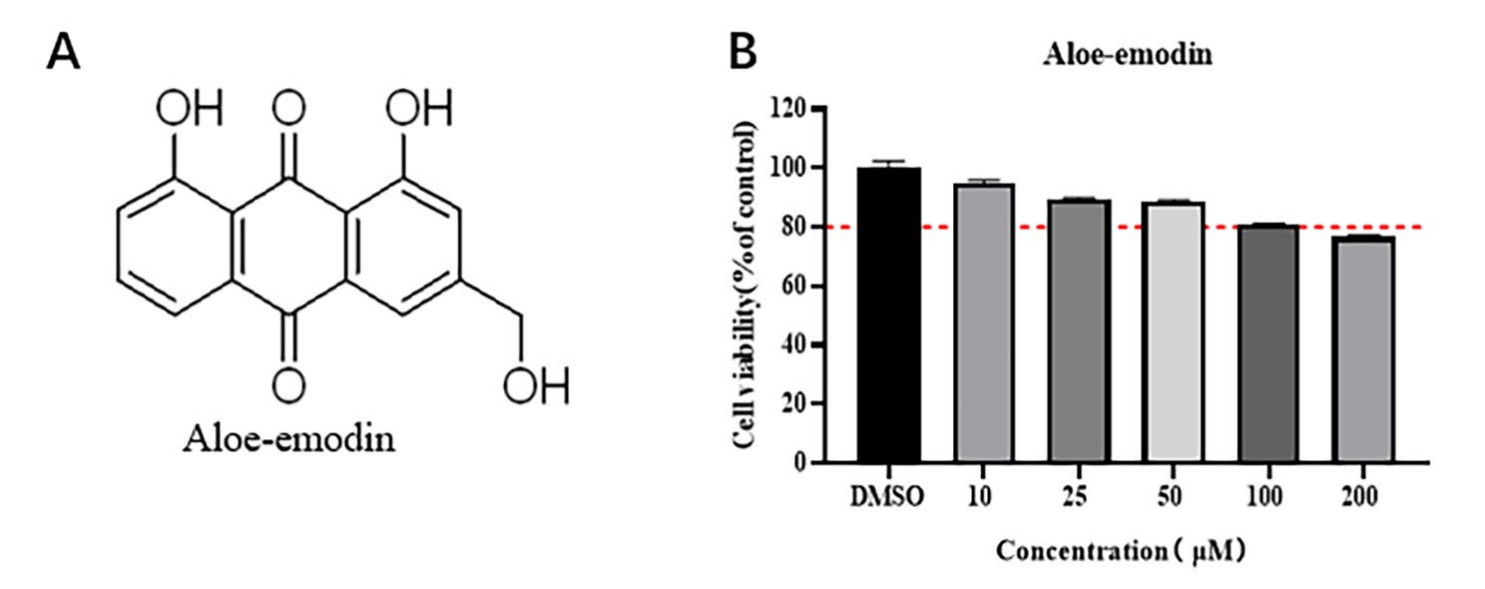
Effects of Ae on PAMs cell viability. (**A**) The chemical structural formula of Ae. (**B**) The toxic effects of Ae on PAMs were detected by CCK-8 assay following incubation with different concentrations of Ae for 48 h. Ae, Aloe-emodin; PAM, porcine alveolar macrophages; CCK-8, cell counting KIT-8. Data were obtained from three independent experiments

According to previous reports, natural plants can be used as novel antiviral agents [10]. *Aloe vera* has a significant inhibitory effect on influenza virus, herpes simplex virus type 1, and other pathogens [11]. In addition to the antiviral effect of the whole aloe extract, the isolated compounds also exhibited antiviral effects. Ae is a natural anthraquinone derivative with the chemical formula C_15_H_10_O_5_ (Fig. 1A). Ae is an active ingredient of Chinese herbs, such as *Cassia occidentalis*, *Rheum palmatum L*., *Aloe vera*, and *Polygonum multiflorum* Thunb [12]. A recent research suggests that Ae has antiviral, anticancer, and anti-inflammatory properties and is involved in immunological modulation and induction of apoptosis [13]. Under inflammatory stress, Ae can inhibit the expression of inducible nitric oxide synthase, degradation of the NF-κB inhibitory IκBα protein, and phosphorylation of ERK, p38, JNK and Akt proteins. In addition, proinflammatory factors in the NF-κB pathway are downregulated to resist inflammation [14]. Furthermore, Ae can significantly increase the level of intracellular reactive oxygen species, inhibit cell proliferation, and induce apoptosis [15].

Although studies have reported that Ae has antiviral effects against porcine reproductive and respiratory syndrome virus (PRRSV) [16], influenza virus (IV) [17], and SARS-CoV [18], its antiviral activity against ASFV and related mechanisms remain unclear. Therefore, the purpose of this study is to use transcriptomic analysis to reveal the changes in the NF-κB signaling pathway and the expression of cytokines IL-1β and IL-8 after PAMs infected by ASFV, as well as the apoptosis of PAMs cells in ASFV. Furthermore, we used Ae to inhibit ASFV replication by promoting apoptosis via blocking NF-κB signaling pathway activated by ASFV infection in vitro, which laid a theoretical foundation for exploring the clinical application of Ae in the prevention and treatment of ASF.

## Materials and methods

### Cell culture and virus

Primary porcine alveolar macrophages (PAMs) were collected from 4-week-old specific pathogen-free pigs. RPMI 1640 medium (Gibco, Waltham, MA, USA) was used to culture PAMs, which supplemented with 100 U/mL penicillin, 50 μg/mL streptomycin, 0.25 μg/mL amphotericin, and 10% fetal bovine serum (FBS) at 37°C in 5% CO_2_. ASFV strain GZ201801 (GenBank: MT496893.1), which is previously isolated in our laboratory, obtained from the Tertiary Animal Biosafety Laboratory of South China Agricultural University.

### Cell viability assay

Cell Counting KIT-8 (CCK-8) (NCM Biotech, Shanghai, China) was used to detect the cytotoxicity of Ae against PAMs. Ae powder was purchased from Bioforte Biotechnology Co.Ltd. (Chengdu Pusi, Chengdu, China). The solution was dissolved to a storage concentration of 100 mM using dimethyl sulfoxide (DMSO). Ae stock solution (100 mM) was diluted with RPMI 1640 medium with 10% FBS to achieve final concentrations of 200, 100, 50, 25 and 10 μM. Different concentrations of Ae were applied to PAMs and incubated at 37°C and 5% CO_2_ for 48 h. Next, 10 μL of the CCK-8 reagent was added to each well and incubated for another 1 h. The absorbance was measured at OD_450_ nm using a microplate reader. The relative viability of the cells was analyzed using the formula:

Cell survival (%)=[OD (drug) -OD (blank)/OD (control) -OD (blank)] ×100%

### ASFV infection and in vitro drug management

The PAMs were spread into 24-well plates. After the cells had completely adhered to the wall, they were infected with 1 multiplicity of infection (MOI) ASFV and treated with 100 μM Ae or 10 μM NF-κB inhibitor (BAY11-7082). The cells were cultured in an incubator with 5% CO_2_ at 37 ℃. At 0, 3, 6, 12, 24, 36 and 48 h after infection, a mixture of cells and the supernatant was collected. After three freeze-thaw cycles, DNA or RNA was extracted for qRCR.

### Detection of apoptosis by flow cytometry

PAMs were plated in 6-well plates. After the cells had completely adhered, they were simultaneously infected with 1 MOI ASFV, treated with 100 μM Ae or 10 μM BAY11-7082, and then cultured at 37°C in 5% CO_2_. After culture for 3, 12 and 48 h, the PAMs were treated with 0.25% trypsin for detach cells from plate. The cells collected were treated with Annexin V-FITC/PI Apoptosis Detection Kit (Beyotime, Shanghai, China). Cell fluorescence was detected using a Beckman flow cytometer, and the results were analyzed using CytExpert software.

### Indirect immunofluorescence assay (IFA)

The PAMs were plated in 48-well plates. After the cells had completely adhered, they were simultaneously infected with 1 MOI ASFV, treated with 100 μM Ae, and then cultured in an incubator at 37°C in 5% CO_2_. The cells were fixed with 4% paraformaldehyde for 30 min at room temperature, and permeabilized with 0.1% Triton-100 for 30 min. The cells were then blocked with 5% bovine serum albumin (BSA) for 1 h and incubated at 37°C. The mouse-derived ASFV p30 protein monoclonal antibody (prepared in our laboratory) and FITC-labeled goat anti-mouse IgG (Beyotime) were used as primary and secondary antibodies, respectively. The aim was to detect the expression of ASFV p30 protein in cells. Nuclei were stained with 4,6-diamino-2-phenylindole (DAPI) and the cells were visualized using a fluorescent inverted microscope (NIKON CORPORATION, Tokyo, Japan).

### Western blot analysis

Cells in the ASFV-infected, drug-treated ASFV-infected and control groups were lysed with RIPA lysis buffer (Beyotime) containing protease and phosphatase inhibitors (Beyotime). The lysed cells were then centrifuged and the supernatant was collected to obtain protein. A BCA protein concentration assay kit (Beyotime) was used to determine total protein concentration. The 5×SDS protein electrophoresis loading buffer (Beyotime) was added to the protein samples, and the protein was denatured by boiling for 15 min. These samples were separated by electrophoresis on 12% sodium dodecyl sulfate polyacrylamide gel electrophoresis (SDS-PAGE), which were then transferred to nitrocellulose (NC) membranes (Merck KGaA, Billerica, MA, USA). The NC membranes were blocked with 5% skim milk for 1 h at 37°C and then incubated with MyD88 (Proteintech, Chicago, IL, USA, 23230-1-AP), pp65 (Cell Signaling Technology, Danvers, MA, USA, 3033S), pIκB (Cell Signaling Technology, Danvers, MA, USA, 9246S), Cleaved Caspase-3 (Cell Signaling Technology, 9664T), Bax (Abcam, Cambridge, UK, ab32503), Bcl-2 (Abcam, Cambridge, UK, ab182858), and α-Tubulin (Beyotime, Shanghai, China, AF0001) antibodies for 1 h at 37°C. NC membranes were incubated with IRDye ® 800CW goat anti-mouse IgG antibody (LI-COR Biosciences, Lincoln, NE, USA) or goat anti-rabbit IgG (LI-COR Biosciences) at 37°C for 45 min. The results were analyzed using an Azure Biosystems infrared imaging system.

### Real-time quantitative PCR (qPCR)

An Axyprep Body Fluid Viral DNA/RNA Miniprep Kit (Axygen, China, AP-MN-BF-VNA) was used to extract whole DNA. The copy number of the ASFV B646L gene was detected using the AceQ Universal U + Probe Master Mix V2 kit (Vazyme, Nanjing, China) and CADC p72 primers and probes. An RNA rapid extraction kit (Fastagen, Shanghai, China) was used to extract Total RNA. The total RNA was reverse-transcribed into cDNA using StarScript 11 First-strand cDNA Synthesis Mix (GenStar, Beijing, China). The ChamQ Universal SYBR qPCR Master Mix kit (Vazyme) and specific primers for IL-1β and IL-8 were used to qPCR. GAPDH was used as the internal reference gene. Relative mRNA expression was calculated using the 2^-ΔΔCT^ method. All experiments were repeated three times. The primers were designed according to the gene sequence in GeneBank database using oligo7 software. qPCR was performed using primers and probes for the ASFV B646L gene recommended by CADC. Primers and probes were prepared by Guangzhou Tian Synthesis by Yihuiyuan company. The gene-specific primer and probe sequences are listed in Table 1.

**Table 1:** The primer sequences used in this study

Gene	Primer Sequence (5’-3’)
CADC-B646L-rPCRF	ATAGAGATACAGCTCTTCCAG
CADC-B646L-rPCRR	GTATGTAAGAGCTGCAGAAC
CADC-B646L-Probe	FAM-TATCGATAAGATTGAT-MGB
B646L-F	TGAAATAAAATGGAAGCCCACAGATC
B646L-R	ACACTGTACAACATTGCGTAAAAGC
GAPDH-F	GCAAAGACTGAACCCACTAATT
GAPDH-R	TTGCCTCTGTTGTTACTTGGAG
IL-1β-F	ACCTGGACCTTGGTTCTCTG
IL-1β-R	CATCTGCCTGATGCTCTTG
IL-8-F	CACTGTGAAAATTCAGAAATCATTGT
IL-8-R	CTTCACAAATACCTGCACAACC

### Transcriptome sequencing analysis

PAMs were infected with ASFV at a MOI of 1 and a negative control was set. Cell samples were collected at 3, 12 and 48 h after infection, and high-throughput RNA sequencing (RNA-Seq) was used to detect the expression of host cell gene transcription profiles. The SOAPnuke filtering software independently developed by BGI was used for filtering to remove reads containing adapter (adapter contamination), reads with unknown base N content greater than 5%, and bases with quality value less than 15. The proportion of these bases in the total reads was greater than 20% of low-quality reads. Raw reads were screened to obtain high-quality de novo transcriptome sequencing data. Differential expression analysis was then performed to identify differentially expressed genes (DEGs) in each treatment group. Single genes with fold change > 2 and Q-value ≤ 0.05 were considered significantly differentially expressed. The Dr.Tom multi-omics data mining system of BGI was used to perform KEGG analysis of the differentially expressed genes and the results were displayed. Functional annotation and pathway analysis of DEGs were performed using Kyoto Encyclopedia of Genes and Genomes (KEGG) database, and the results were presented.

### Statistical analysis

According to the existing transcriptomic data of ASFV-infected PAMs in our laboratory, functional annotation of differentially expressed genes (DEGs) at three time points of ASFV infection was performed using KEGG pathway enrichment analysis. All data are presented as mean ± standard error (SE) of three independent experiments. Results were subjected to a one-way analysis of variance (ANOVA) by GraphPad Prism 8.0.1 software (San Diego, CA, USA), which followed by Tukey’s t-test. * P < 0.05, ** P < 0.01, and *** P < 0.001 were set as Statistical significance.

## Results

### The effect of Ae on the activity of PAMs

The CCK-8 assay was used to detect the potential cytotoxic effect of Ae on PAMs at concentrations of 10, 25, 50, 100 and 200 μM. As shown in Fig. 1B, when cells were incubated with different concentrations of Ae for 48 h, the viability of PAMs decreased in a dose-dependent manner compared to the control group. When treating PAMs with 200 μM Ae, cell viability dropped below 80% (Fig. 1B). Based on the maximum safe concentration of Ae in PAMs, Ae at a concentration of 100 μM was selected for subsequent experiments.

### Ae inhibits the replication level of ASFV

**Fig. 2:**
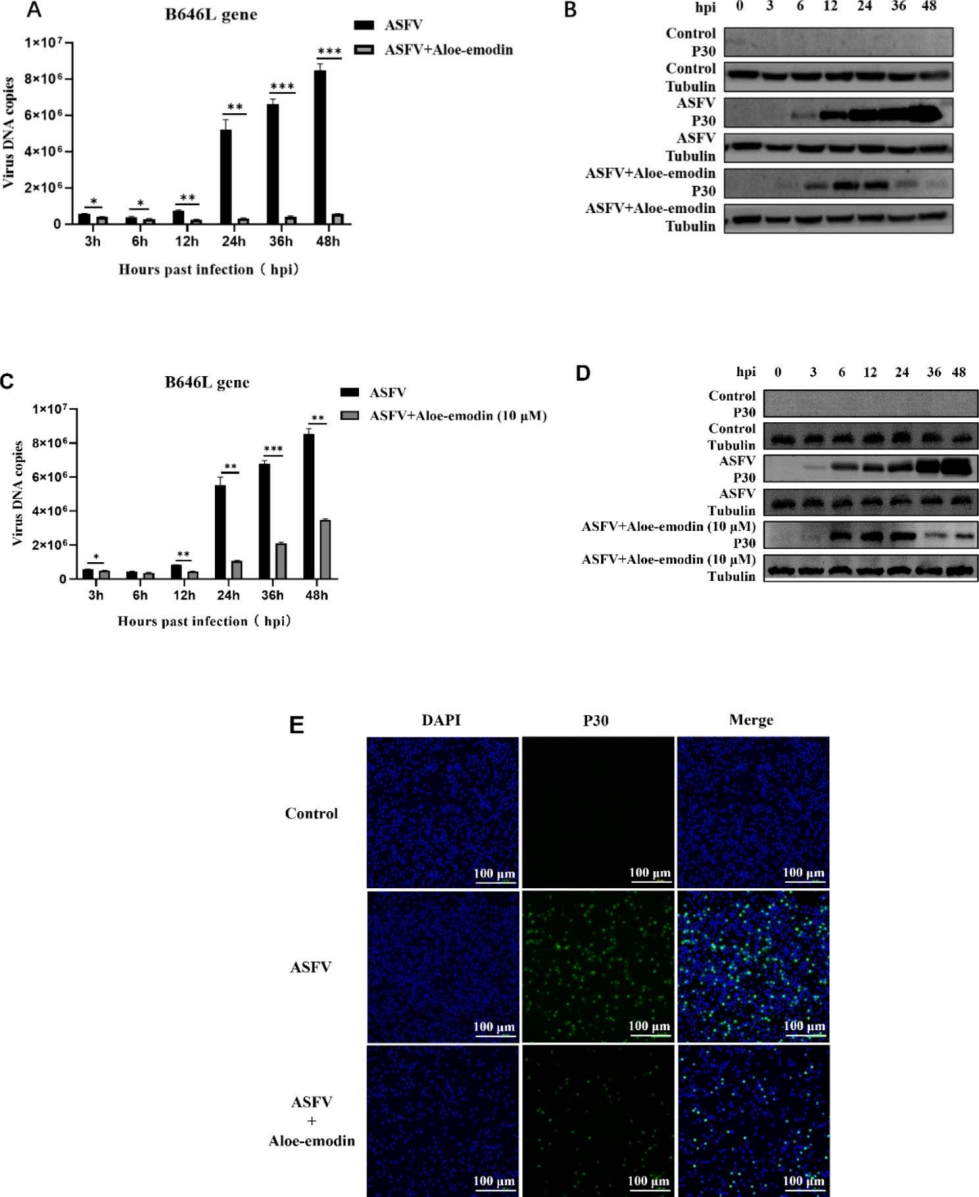
Antiviral activity of Ae against ASFV (**A** and **C**) The ASFV B646L gene copy-number in the ASFV-infected group and Ae-treated ASFV-infected group was detected at different time points by qPCR. The results of three independent experiments (mean ± SD) were represented by one data. (**B** and **D**) The ASFV p30 protein expression level in the ASFV-infected group or Ae-treated ASFV-infected group were detected by western blotting. (**E**) IFA was used to detect the expression level of p30 protein in the control group, the ASFV-infected group and the Ae-treated ASFV-infected group, and the antiviral activity of Ae against ASFV was determined. The results of three independent experiments (mean ± SD) were represented by one data. The graph above is representative of three independent IFA trials with similar results. Significant differences that were compared to the control group were indicated by * (P < 0.05), ** (P < 0.01) and *** (P < 0.001). ASFV, African swine fever virus; qPCR, real-time polymerase chain reaction; IFA, Indirect Immunofluorescence Assay

The effect of Ae on ASFV replication in vitro was studied using qPCR. Compared with the ASFV-infected groups at 12, 24, 36 and 48 h, the ASFV B646L copy-number significantly decreased after treated with 10 μM or 100 μM Ae (Fig. 2A, 2C). However, treatment with 100 μM Ae was more effective than that with 10 μM Ae. To further determine whether Ae inhibits the replication of ASFV, we determined the expression of ASFV p30 protein by western blotting and an indirect immunofluorescence assay. The results of western blotting showed that with an increase in the infection time, the p30 protein expression also increased significantly in the group of ASFV-infected. The expression level of the ASFV p30 protein was markedly decreased in the 10 μM or 100 μM Ae-treated group by comparison to that in the ASFV-infected group (Fig. 2B, 2D). The IFA results showed that compared against the 48 h ASFV-infected group, the Ae-treated group had significantly less ASFV p30 protein fluorescence than the group of ASFV-infected cells (Fig. 2E). These results indicate that Ae has an anti-ASFV activity in vitro.

### Transcriptomic analysis

**Fig. 3:**
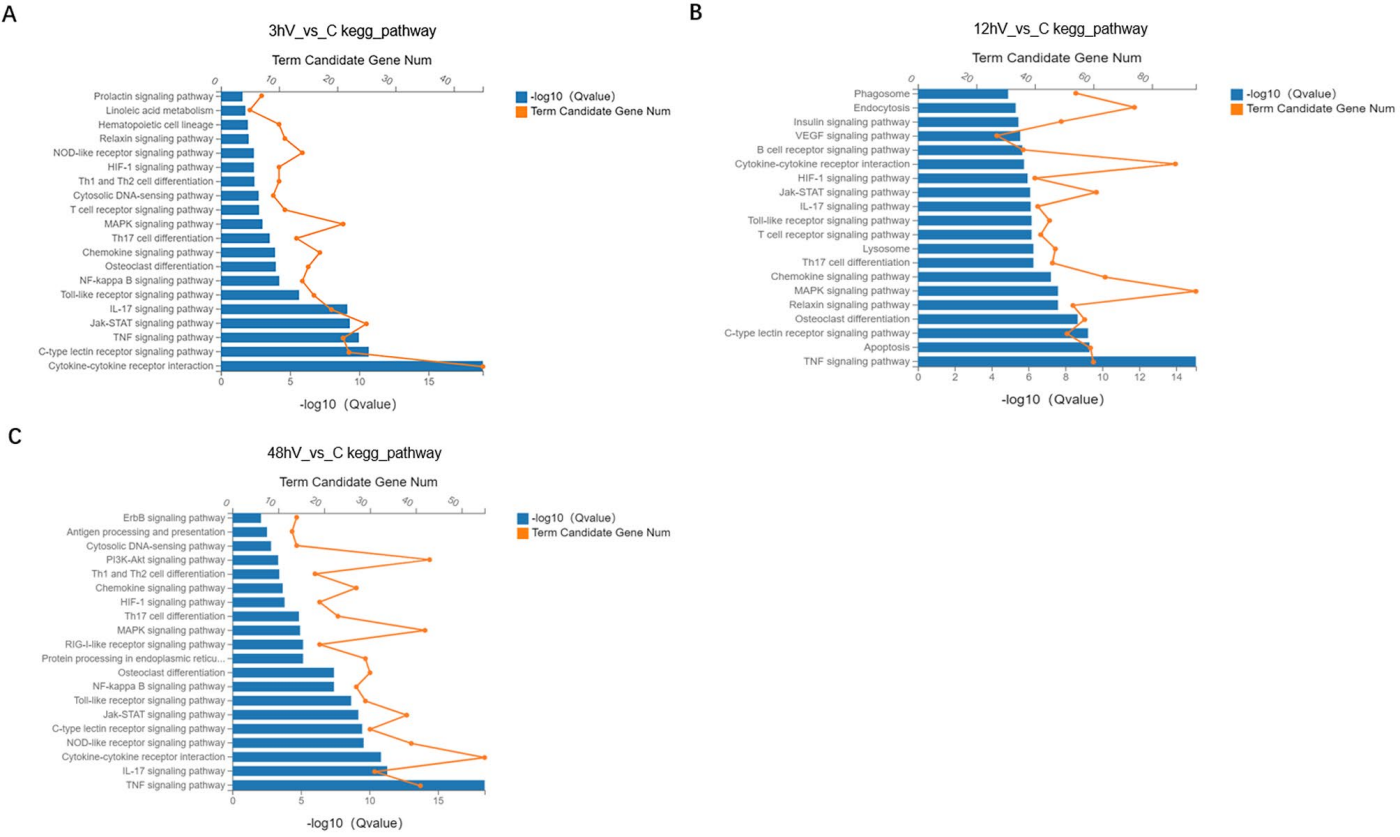
KEGG pathway enrichment analysis of DEGs in ASFV infection group and control group at 3, 12 and 48 h. KEGG pathway enrichment analysis was performed on differentially expressed genes between the infection group and the control group following 1 MOI infection of PAMs for (**A**) 3 h, (**B**) 12 h, and (**C**) 48 h. DEG, differentially expressed genes

For exploring how ASFV regulates host gene transcription, ASFV-infected PAMs were subjected to transcriptomic analysis. PAMs were infected with ASFV at 1 MOI, and the cells were collected at 3, 12 and 48 h after infection, and total RNA was extracted from each group of cells and subjected to genomic analysis. A negative control group was set at each time point, and RNA isolated from living cells in each group subjected to RNA and amino acid sequencing. DEGs in the control and infected groups were subjected to KEGG pathway enrichment analysis. At 3 and 48 h of ASFV infection, the differential genes were mainly enriched in the NOD-like receptor signaling pathway, osteoclast differentiation, NF-κB signaling pathway, chemokine signaling pathway, Toll-like receptor signaling pathway, IL-17 signaling pathway, TNF signaling pathway, cytokine-cytokine receptor interaction, MAPK signaling pathway, C-type lectin receptor signaling pathway and JAK-STAT signaling pathway (Fig. 3A, B). The DEGs at 12 h of ASFV infection were mainly enriched in endocytosis, apoptosis, chemokine signaling pathway, MAPK signaling pathway, cytokine-cytokine receptor interaction, Jak-STAT signaling pathway and TNF signaling pathway (Fig. 3C). It has been demonstrated that Ae can prevent nerve injury and neuroinflammation caused by ischemic stroke via inhibiting the NF-κB signaling pathway [19]. In addition, Ae and emodic-acid inhibited NF-κB activity in breast cancer cells and proliferation of breast cells [20]. Combined with the transcriptomic analysis results, we selected the NF-κB signaling pathway for further study on how Ae regulates the NF-κB signaling pathway against ASFV.

### Ae inhibits the NF-κB signaling pathway activated by ASFV infection

**Fig. 4:**
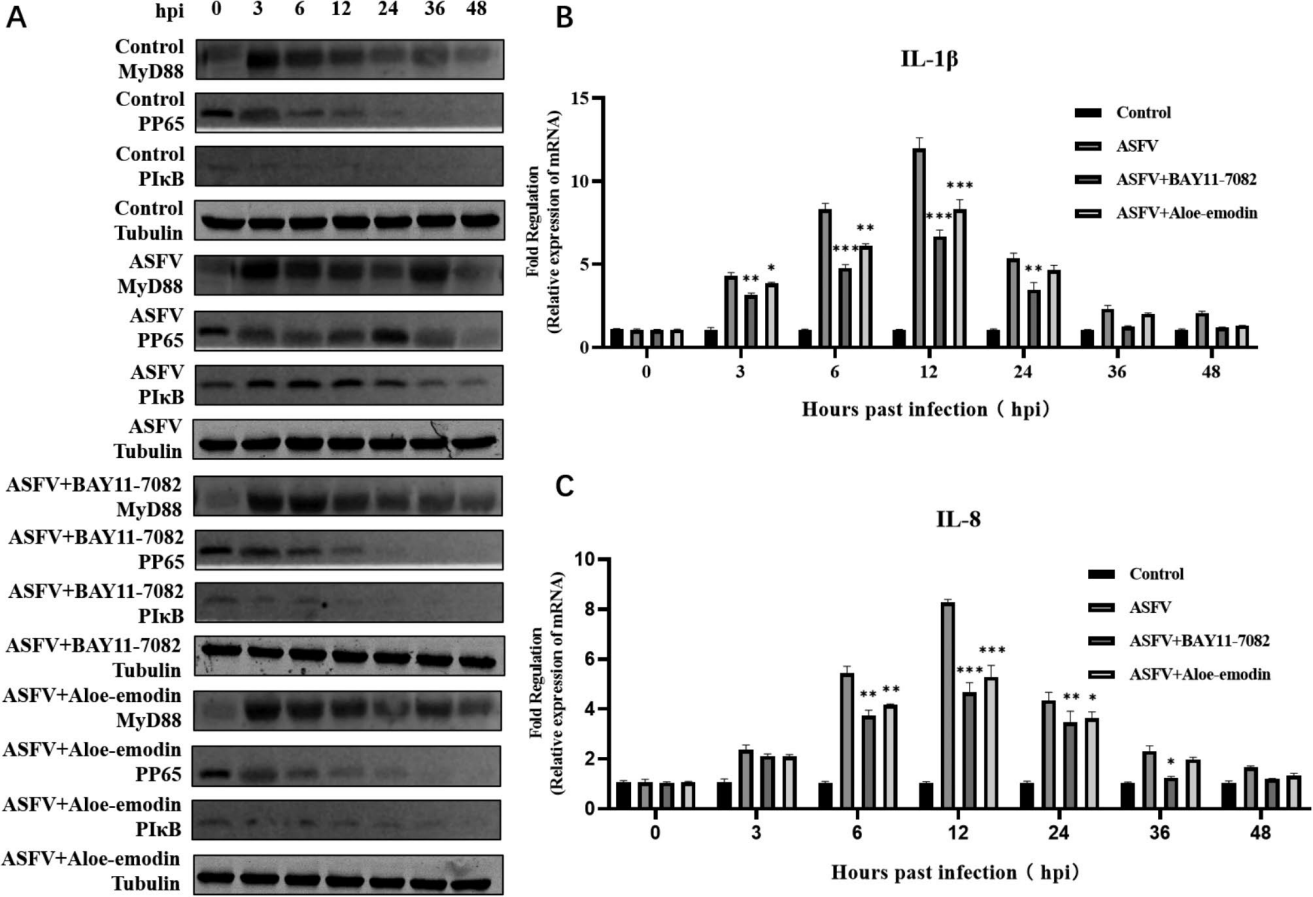
Ae inhibits the NF-κB signaling pathway activated by ASFV infection. (**A**) The expression level of MyD88 protein, phospho-NF-κB p65 protein, and pIκB protein in the ASFV-infected group, ASFV-infected group treated with BAY11-7082 or Ae was detected by western blot. The expression of tubulin was used as a positive control. qPCR was used to detect the changes of (**B**) IL-1β mRNA levels and (**C**) IL-8 mRNA levels in the ASFV-infected group, ASFV-infected group treated with BAY11-7082, or Ae at different time points. The mRNA level of GAPDH was used as a positive control. All control cells were normal cultured PAMs. The results of three independent experiments (mean ± SD) were represented by one data. Significant differences that were compared to the control group were indicated by * (P < 0.05), ** (P < 0.01) and *** (P < 0.001)

It has been reported that the NF-κB signaling pathway was activated by ASFV infection, and the NF-κB signaling pathway inhibitor can inhibit ASFV replication [21]. We further explored the effect of Ae on the activation of the NF-κB signaling pathway in ASFV infection. In this study, changes in protein expression levels in the NF-κB signaling pathway were detected by western blotting experiments at 3, 6, 12, 24, 36 and 48 h after 1 MOI ASFV infection of PAMs. The results showed that the protein expression levels of MyD88, phospho-NF-κB p65, and pIκB were significantly increased in the ASFV-infected group by compared with the control group. In comparison to the ASFV-infected group, MyD88, phospho-NF-κB p65, and pIκB (Fig. 4A) protein expression levels were significantly decreased in the ASFV-infected group treated with BAY11-7082 or Ae. It has been reported that the mRNA levels of IL-1β and IL-8 significantly increase after ASFV infection, and treatment with an NF-κB inhibitors significantly inhibited the mRNA levels of IL-1β and IL-8, which were upregulated by ASFV infection [21]. Therefore, we explored the mRNA levels of IL-1β and IL-8 in the ASFV-infected group treated with Ae. qPCR revealed that Ae significantly inhibited the mRNA levels of NF-κB signaling pathway-related inflammatory factors IL-1β (Fig. 4B) and IL-8 (Fig. 4C), which were upregulated by ASFV infection. These results indicate that Ae downregulates the NF-κB signaling pathway which is activated by ASFV infection.

### Ae promotes apoptosis by inhibiting the NF-κB signaling pathway

**Fig. 5:**
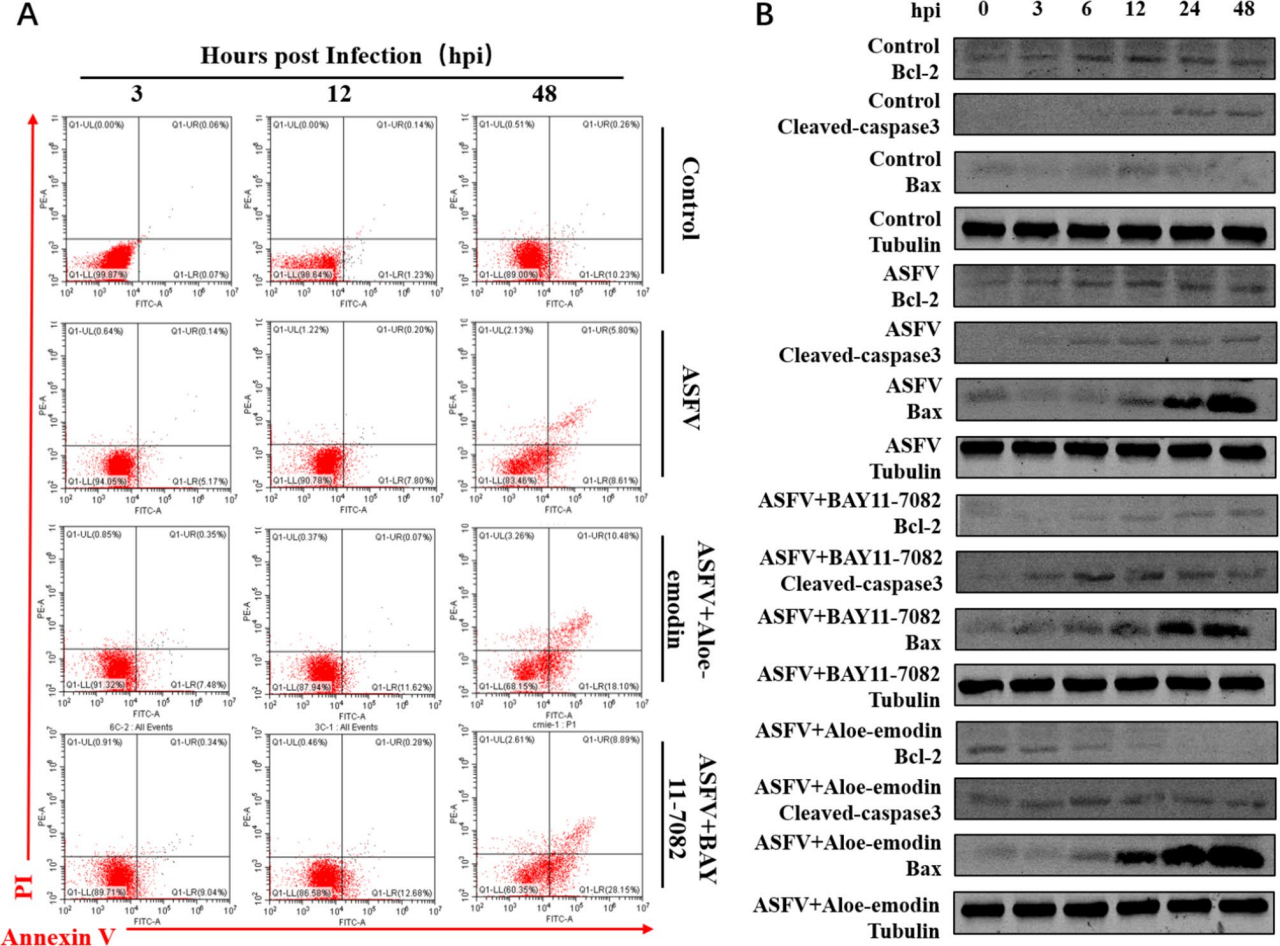
Ae promotes apoptosis by inhibiting the NF-κB signaling pathway. (**A**) The apoptosis of PAMs in the ASFV infection group, BAY11-7082 or Ae-treated ASFV infection group was detected by flow cytometry, and the apoptosis of induced cells was detected at 3, 12 and 48 h after treatment. Untreated cells served as negative controls. The expression level of (**B**) Bcl-2 protein, cleaved-caspase3 protein, and Bax protein in the ASFV infection group, BAY11-7082 or Ae-treated ASFV infection group was detected by western blot. All control cells were normal cultured PAMs. The tubulin expression was used as a positive control

To explore the effect of Ae on the NF-κB signaling pathway during apoptosis, ASFV-infected PAMs were treated with BAY11-7082, an inhibitor of the NF-κB signaling pathway, as a positive control. PAMs infected with 1 MOI ASFV were treated with Ae or BAY11-7082, and the effect of Ae or BAY11-7082 on ASFV infection-induced apoptosis of PAMs was detected using flow cytometry. The percentage of apoptotic cells in the ASFV-infected group treated with Ae or BAY11-7082 was markedly higher than that in the control and ASFV-infected groups (Fig. 5A). The expression levels of apoptosis-related proteins were detected by western blotting, and Ae was shown to inhibit the expression levels of anti-apoptotic protein Bcl-2 (Fig. 5B) and promote the expression of cleaved-caspase3 and pro-apoptotic protein Bax, thereby promoting ASFV infection-induced apoptosis. These results indicate that Ae promotes apoptosis by inhibiting the NF-κB signaling pathway.

## Discussion

ASFV has continued to spread globally in the swine industry since it was first reported. In recent years, the unprecedented spread of African swine fever has caused serious economic losses to the pig industry worldwide [22]. Therefore, controlling the spread of ASF has become a top priority in the pig industry. In addition to the urgent need for research and development of ASFV vaccines, the use of natural and effective compounds to develop potent anti-ASFV drugs has also become a current research hotspot. Although many effective anti-ASFV drugs have been reported, the antiviral mechanisms of some of these compounds remain unclear. Therefore, an in-depth study of the molecular mechanisms of drugs against ASFV infection in the host is significant for the development of effective antiviral drugs. Our previous research found that ASFV infection activates the NF-κB signaling pathway at an early stage, and that an NF-κB inhibitors can significantly inhibit the replication of ASFV [21]. In this research, transcriptomic analysis of ASFV-infected PAMs revealed that infection with ASFV-GZ201801 activates the NF-κB signaling pathway at the early stage and the apoptosis pathway at a later stage. At the same time, we found that Ae can inhibit ASFV replication via blocking the NF-κB signaling pathway and promoting apoptosis, which helps control the spread of ASFV infection in pig farms.

In recent years, natural plants have been widely used as raw materials for new antiviral drugs owing to their relatively minor side effects, abundance, and low cost [23]. Ae is an anthraquinone compound with various pharmacological effects, including antiviral, anti-inflammatory, immunosuppressive, and anticancer effects [13]. It has been reported to inhibit the replication of porcine epidemic diarrhea virus (PEDV) both in vivo and in vitro [24]. Simultaneously, Ae can inhibit the replication of porcine reproductive and respiratory syndrome virus (PRRSV) by activating Toll-like receptor 3 [17]. These findings prompted us to explore whether Ae effectively inhibits ASFV replication in vitro, which is consistent with our findings. In addition, further studies on the antiviral molecular mechanism of Ae in regulating the interaction between ASFV and the host is imperative.

Apoptosis, also known as programmed cell death, is a process of cell death caused by triggering the death program in cells both in vitro and in vivo [25]. There are two main apoptosis pathways: the extracellular death receptor pathway and the intracellular mitochondrial pathway [26]. The former mainly activates genes such as Bcl-2 and NF-κB in cells by stimulating factors, while the latter affects the membrane potential of mitochondria in cells through stimulating factors. Both pathways ultimately lead to caspase-3 activation [27–29]. These studies have shown that ASFV infection upregulates the expression of proinflammatory cytokines, and BAY11-7082 can regulate ASFV-induced inflammatory responses by downregulating the expression of proinflammatory cytokines [22]. NF-κB plays a key role in maintaining physiological and normal immune functions [30]. It mediates biological processes such as the inflammatory response, apoptosis, cell proliferation, and immune response by activating the transcription of many target genes [31]. In recent years, studies have shown that NF-κB is closely related to apoptosis, is involved in the transcriptional regulation of various apoptosis-related genes, and has bidirectional effects in inhibiting and promoting apoptosis [32–35]. Numerous studies have shown that various antiviral drugs, including breviscapine [36], baicalein [17], curcumin [37], and hypericin [38] can promote apoptosis by inhibiting the NF-κB pathway. The NF-κB signaling pathway can induce apoptosis by affecting the protein expression levels of cleaved-caspase3, Bcl-2, and Bax [37]. Therefore, it is crucial to study the effect of ASFV on viral replication by regulating the NF-κB signaling pathway to induce apoptosis. We confirmed that Ae inhibits ASFV proliferation by promoting cell apoptosis, and speculated that the reason is that the virus needs the host as a replication site, and it is difficult for the virus to continue to proliferate after a large number of cell apoptosis. Hence, Ae exerts an antiviral effect by inducing massive apoptosis of ASFV-infected PAMs, thereby reducing the replication site of ASFV.

In the present study, we found that Ae inhibited the replication of ASFV and protein expression levels of MyD88, pIκB, and pp65 in ASFV-infected PAMs. Moreover, Ae inhibits the ASFV-activated NF-κB signaling pathway and downregulates the mRNA levels of the proinflammatory cytokines IL-1β and IL-8. In addition, Ae can inhibit the expression of anti-apoptotic protein Bcl-2 and promote the expression level of cleaved-caspase3 and proapoptotic protein Bax, thereby promoting the apoptosis of ASFV-infected PAMs. However, whether Ae can inhibit ASFV infection in vivo remains unknown.

## Conclusions

**Fig. 6:**
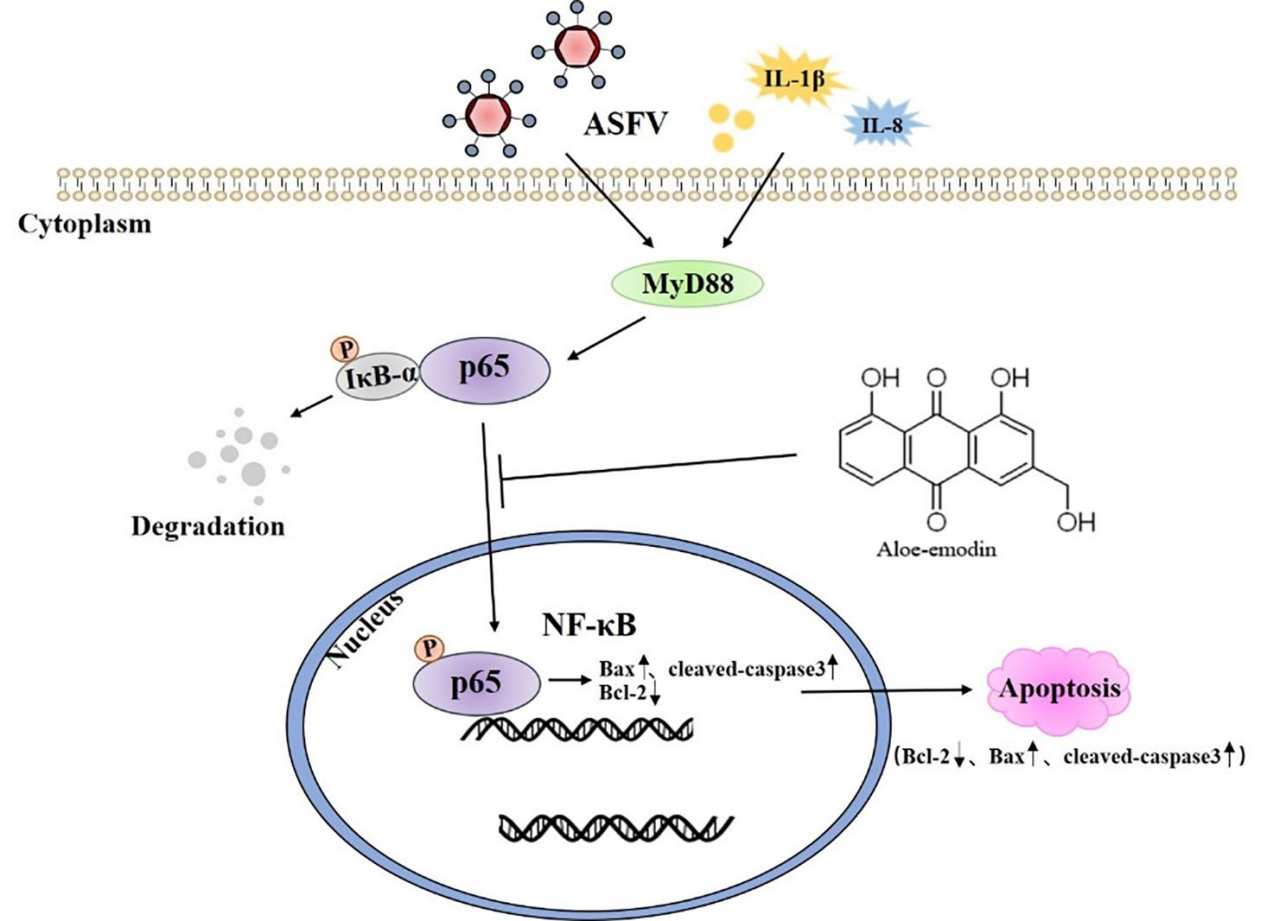
Aloe-emodin can promote apoptosis and inhibit ASFV replication via blocking the NF-κB signaling pathway activated by ASFV infection. After infection with PAMs, GZ201801-ASFV promotes the release of IL-1β and IL-8 to induce MyD88 expression. Aloe-emodin treatment of ASFV-infected PAMs significantly blocked MyD88-induced NF-κB activation to promote apoptosis and inhibit ASFV replication

Our experimental data demonstrated that Ae extracted from natural plants can inhibit ASFV replication in vitro and can promote cell apoptosis by inhibiting the NF-κB signaling pathway activated by ASFV infection, thereby exerting its antiviral effect (Fig. 6). In summary, Our study findings lay a theoretical foundation for the prevention and control of ASFV by providing substantial evidence for the use of Ae in the development of novel anti-ASFV therapies.

## Data Availability

Data is contained within the article material.
