# Correction: Aloe-emodin inhibits African swine fever virus replication by promoting apoptosis via regulating NF-κB signaling pathway

**DOI:** 10.1186/s12985-025-02676-z

**Published:** 2025-03-06

**Authors:** Yizhuo Luo, Yunlong Yang, Wenru Wang, Qi Gao, Ting Gong, Yongzhi Feng, Dongdong Wu, Xiaoyu Zheng, Guihong Zhang, Heng Wang

**Affiliations:** 1 Key Laboratory of Zoonosis Prevention and Control of Guangdong Province, College of Veterinary Medicine, South China Agricultural University, Guangzhou, 510462 China; 2 African Swine Fever Regional Laboratory of China (Guangzhou), Guangzhou, 510642 China; 3 Key Laboratory of Animal Vaccine Development, Ministry of Agriculture and Rural Affairs, Guangzhou, PR China; 4 Maoming Branch, Guangdong Laboratory for Lingnan Modern Agriculture, Maoming, 525000 China; 5 Research Center for African Swine Fever Prevention and Control, South China Agricultural University, Guangzhou, 510642 China

**Correction to: Virology Journal (2023) 20:158** 10.1186/s12985-023-02126-8

In this article [1], Fig(s) 4 and 5 appeared incorrectly and have now been corrected in the original publication. For completeness and transparency, both the incorrect and correct versions are displayed below.

Incorrect Figs. 4 and 5

**Fig. 4:**
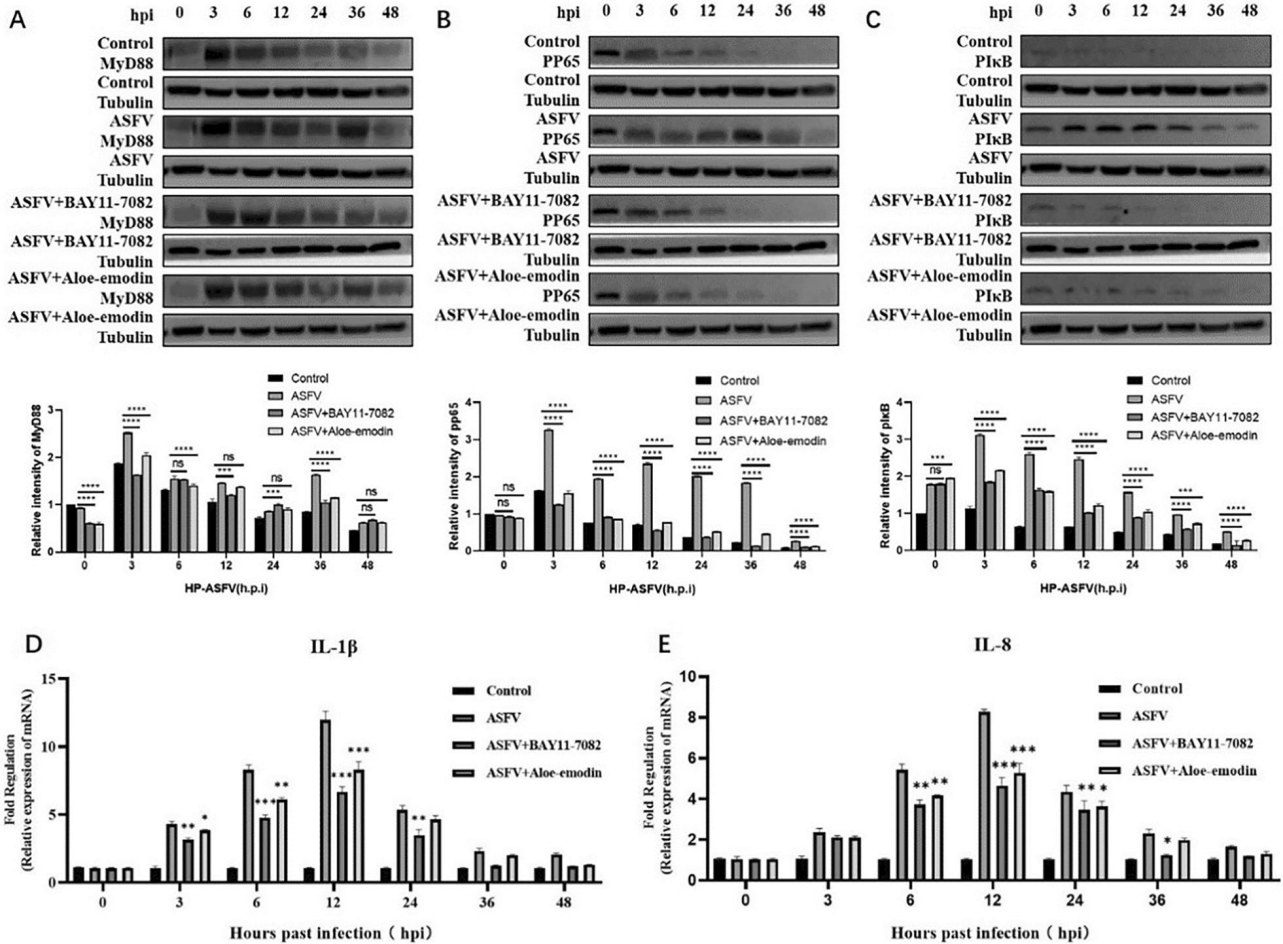
Ae inhibits the NF-κB signaling pathway activated by ASFV infection. The expression level of (**A**) MyD88 protein, (**B**) phospho-NF-κB p65 protein, and (**C**) pIκB protein in the ASFV-infected group, ASFV-infected group treated with BAY11-7082 or Ae was detected by western blot. The expression of tubulin was used as a positive control. qPCR was used to detect the changes of (**D**) IL-1β mRNA levels and (**E**) IL-8 mRNA levels in the ASFV-infected group, ASFV-infected group treated with BAY11-7082, or Ae at different time points. The mRNA level of GAPDH was used as a positive control. All control cells were normal cultured PAMs. The results of three independent experiments (mean ± SD) were represented by one data. Significant differences that were compared to the control group were indicated by * (P < 0.05), ** (P < 0.01) and *** (P < 0.001)

**Fig. 5:**
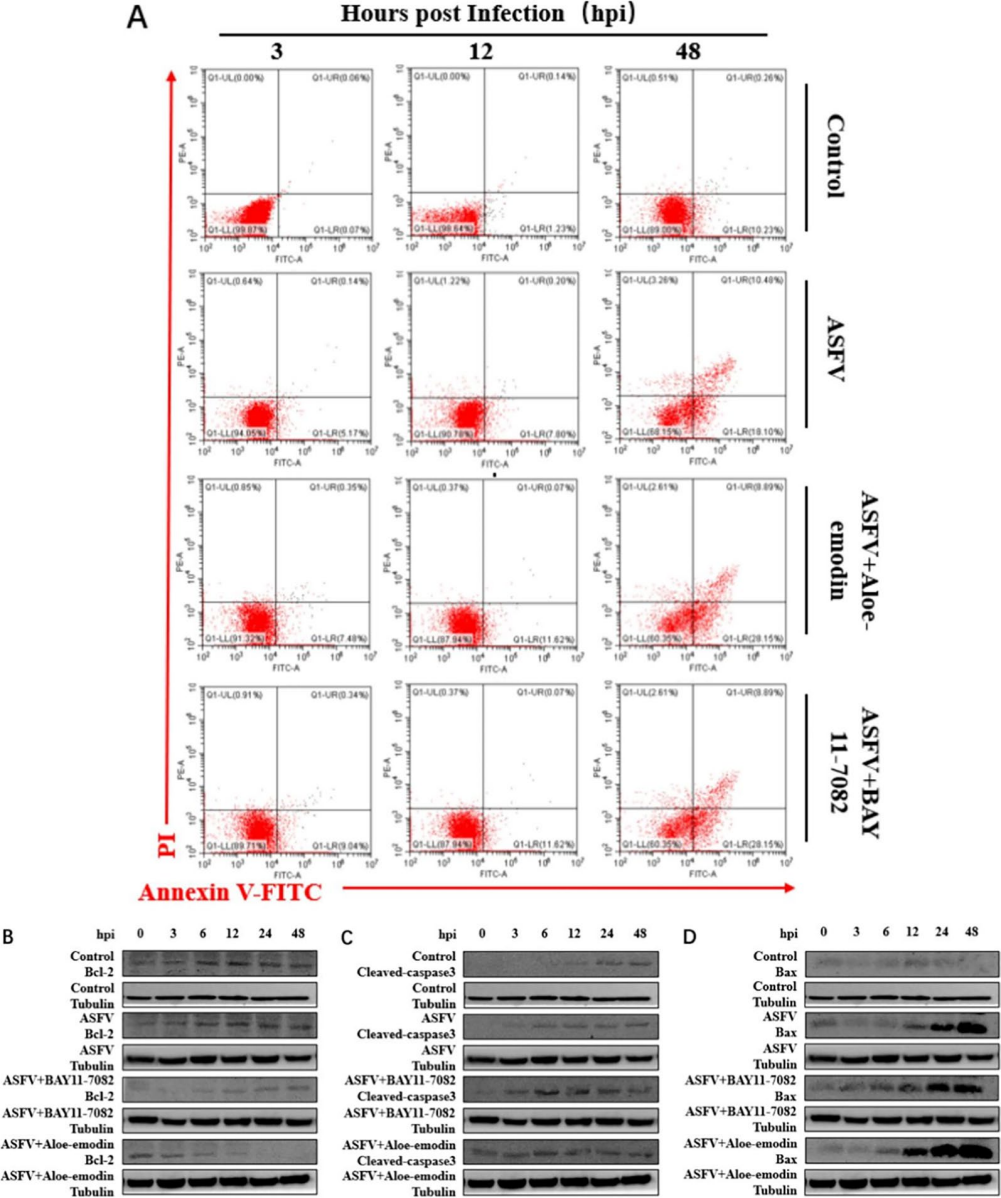
Ae promotes apoptosis by inhibiting the NF-κB signaling pathway. (**A**) The apoptosis of PAMs in the ASFV infection group, BAY11-7082 or Ae-treated ASFV infection group was detected by flow cytometry, and the apoptosis of induced cells was detected at 3, 12 and 48 h after treatment. Untreated cells served as negative controls. The expression level of (**B**) Bcl-2 protein, (**C**) cleaved-caspase3 protein, and (**D**) Bax protein in the ASFV infection group, BAY11-7082 or Ae-treated ASFV infection group was detected by western blot. All control cells were normal cultured PAMs. The tubulin expression was used as a positive control

Correct Figs. 4 and 5

**Fig. 4:**
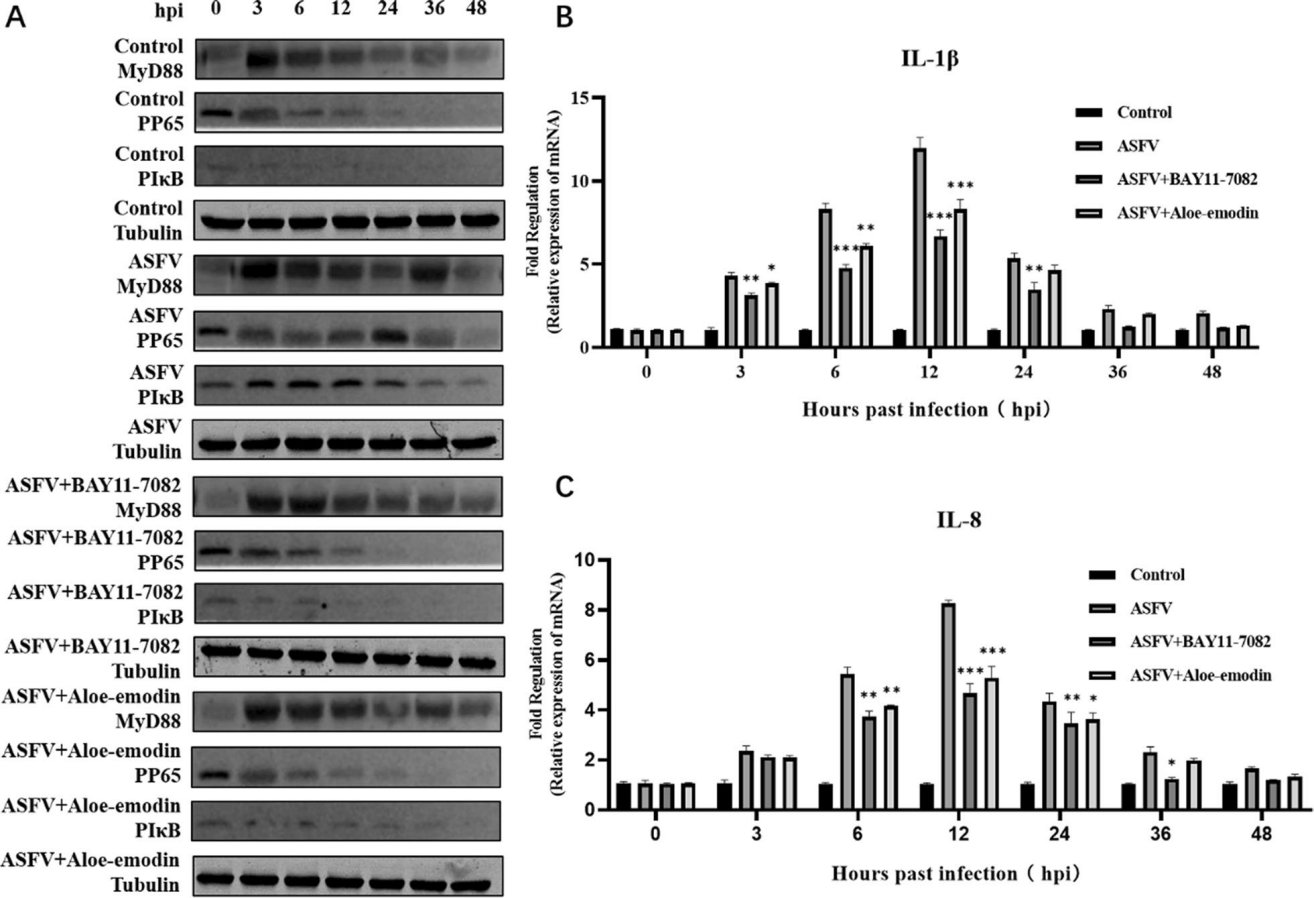
Ae inhibits the NF-κB signaling pathway activated by ASFV infection. **A** The expression level ofMyD88 protein, phospho-NF-κB p65 protein, and pIκB protein in the ASFV-infected group, ASFV-infected group treated with BAY11-7082 or Ae was detected by western blot. The expression of tubulin was used as a positive control. qPCR was used to detect the changes of **B** IL-1β mRNA levels and **C** IL-8 mRNA levels in the ASFV-infected group, ASFV-infected group treated with BAY11-7082, or Ae at different time points. The mRNA level of GAPDH was used as a positive control. All control cells were normal cultured PAMs. The results of three independent experiments (mean ± SD) were represented by one data. Significant differences that were compared to the control group were indicated by * (P < 0.05), ** (P < 0.01) and *** (P < 0.001)

**Fig. 5:**
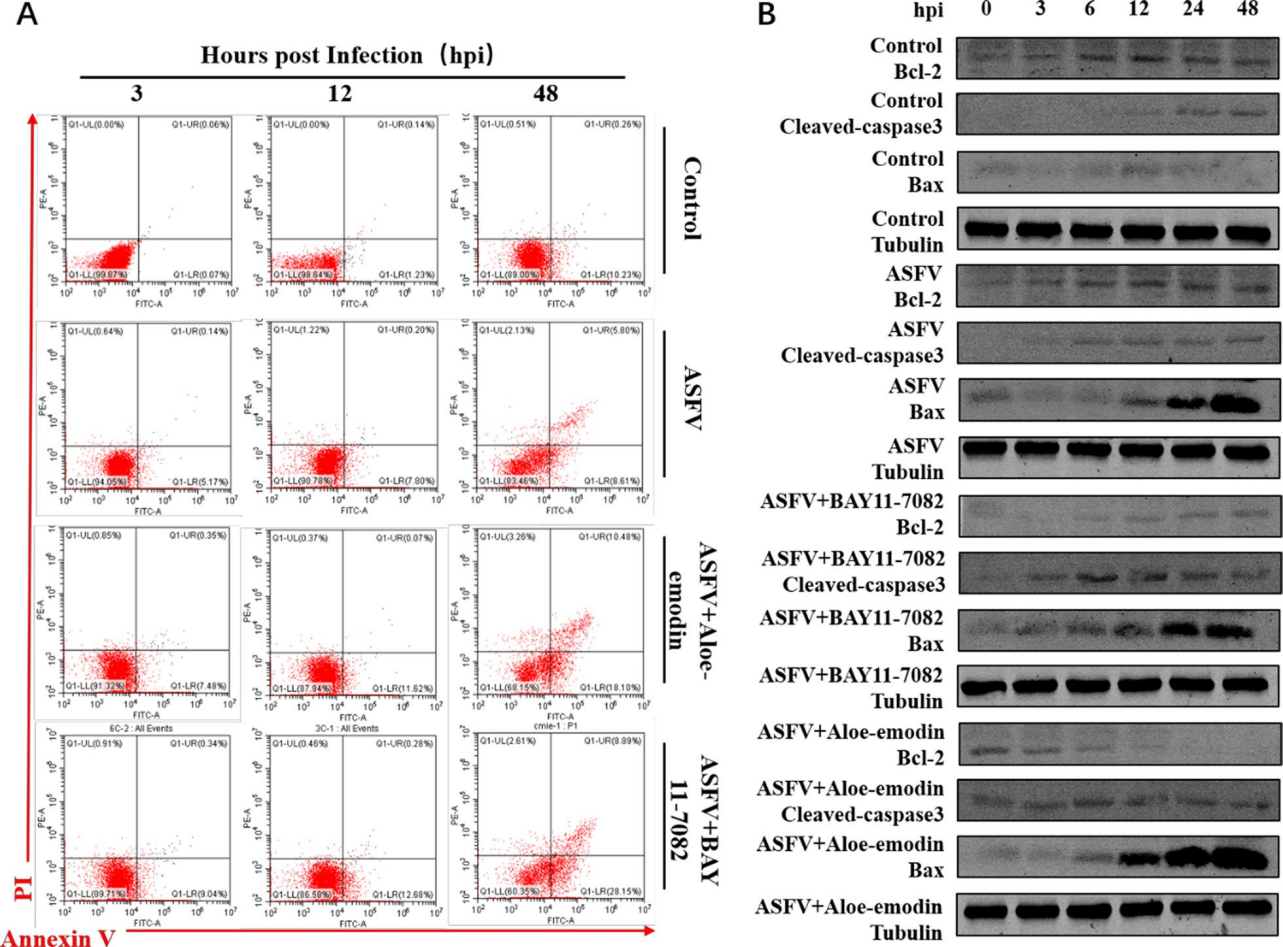
Ae promotes apoptosis by inhibiting the NF-κB signaling pathway. **A** The apoptosis of PAMs in the ASFV infection group, BAY11-7082 or Ae-treated ASFV infection group was detected by flow cytometry, and the apoptosis of induced cells was detected at 3, 12 and 48 h after treatment. Untreated cells served as negative controls. The expression level of **B** Bcl-2 protein, cleaved-caspase3 protein, and Bax protein in the ASFV infection group, BAY11-7082 or Ae-treated ASFV infection group was detected by western blot. All control cells were normal cultured PAMs. The tubulin expression was used as a positive control
